# Parametric Optimization of Lateral NIPIN Phototransistors for Flexible Image Sensors

**DOI:** 10.3390/s17081774

**Published:** 2017-08-02

**Authors:** Min Seok Kim, Gil Ju Lee, Hyun Myung Kim, Young Min Song

**Affiliations:** School of Electrical Engineering and Computer Science (EECS), Gwangju Institute of Science and Technology (GIST), 123, Chemdangwagi-ro, Buk-gu, Gwangju 61005, Korea; seok9643@gmail.com (M.S.K.); gjlee0414@gist.ac.kr (G.J.L.); gusaud31@gist.ac.kr (H.M.K.)

**Keywords:** lateral phototransistor, bio-inspired image sensor, curved photodetector array, flexible/stretchable electronics

## Abstract

Curved image sensors, which are a key component in bio-inspired imaging systems, have been widely studied because they can improve an imaging system in various aspects such as low optical aberrations, small-form, and simple optics configuration. Many methods and materials to realize a curvilinear imager have been proposed to address the drawbacks of conventional imaging/optical systems. However, there have been few theoretical studies in terms of electronics on the use of a lateral photodetector as a flexible image sensor. In this paper, we demonstrate the applicability of a Si-based lateral phototransistor as the pixel of a high-efficiency curved photodetector by conducting various electrical simulations with technology computer aided design (TCAD). The single phototransistor is analyzed with different device parameters: the thickness of the active cell, doping concentration, and structure geometry. This work presents a method to improve the external quantum efficiency (EQE), linear dynamic range (LDR), and mechanical stability of the phototransistor. We also evaluated the dark current in a matrix form of phototransistors to estimate the feasibility of the device as a flexible image sensor. Moreover, we fabricated and demonstrated an array of phototransistors based on our study. The theoretical study and design guidelines of a lateral phototransistor create new opportunities in flexible image sensors.

## 1. Introduction

Imaging systems incorporating bioinspired designs can provide many advantages compared to the conventional imaging devices [1,2,3,4,5]. Mimicking hemispherical retinas or ommatidia of animals is a core concept in bio-inspired imaging systems. A curved image sensor, which is analogous to the retina, can provide a simple configuration and a small volume of the optical system, while maintaining high optical performance [6,7]. Many researchers have reported various approaches to fabricate a curved image sensor, based on advancements of fabrication techniques and materials. Examples include a mechanically curved commercial image sensor [8,9,10,11], a photodetector array based on organic/nanomaterials [12,13,14,15,16], and the use of structured Si that is deformable in 3D [1,2,3,17]. Among them, Si-based electronics have inherent advantages such as an abundance of Si, mature technology, stability, and optical/electrical superiority. On this basis, Si-based flexible electronic devices are not limited to image sensors but are being studied for use in various flexible and wearable devices such as solar cells/photodiodes [18,19,20], tactile/pressure/temperature sensors [21,22], and health monitors [23,24].

Fundamentally, bulk Si can be easily fractured and fail due to its brittleness and rigidity. To secure the flexibility of Si, the thickness of the Si layer should be under tens of micrometers [25,26]. In addition to the use of thin Si, geometrical optimization including the electrodes has been intensely conducted for a large radius of curvature and a high density of photodetector arrays accompanying mechanical stability [27,28]. In these studies, many authors discussed the superiority and strength of the curved photodetector. Although a NIP photodiode (PD) [17,29] or a NIPINphototransistor (PTR) [1,2,3] has been widely used as a single pixel of a curved photodetector array, optimization or comparison of these single pixels has not been conducted. As the curved image sensor consists of thin Si devices, the absorption efficiency of the devices is inherently limited by the film-type active cell. Therefore, device optimization with respect to electronics is necessary for flexible and curved electronics.

Here, we present an optimized single pixel in terms of electrical aspects based on theoretical analyses. In this study, we compared a lateral NIP PD and a NIPIN PTR with the same doping concentration and thickness. From this comparison, we established that the phototransistor is a promising element as the single cell of curved photodetector arrays. To investigate the effects of other parameters with respect to semiconductor electronics, we performed a quantitative analysis of the thickness of the active cell, the doping concentration, and the structural geometry. To verify the validity on our analyses, we fabricated and measured phototransistor arrays that are designed based on our simulation. Also, we present simulations of the passive matrix characteristics of the photodetector array. Finally, we carry out a geometrical study of a single cell to enhance the cell efficiency and the mechanical stability. All simulations in our paper are based on uniform doping distribution and a carrier concentration of pure silicon (10^10^ cm^−3^) for reducing required computational memory and time.

## 2. Simulation and Optimization of Single Device

### 2.1. Comparison between Photodiode and Phototransistor

**Figure 1:**
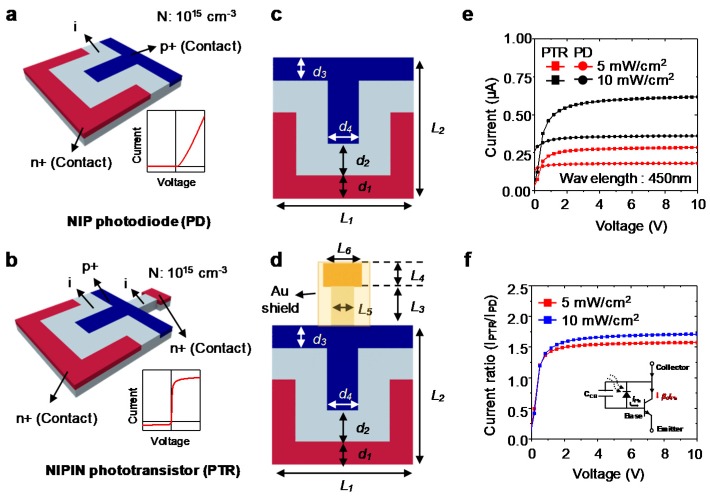
(**a**,**b**) 3D schemes of lateral photodiode and phototransistor. Insets show general I-V curves of photodiode and phototransistor, respectively; (**c**,**d**) geometrical parameters of lateral photodiode and phototransistor. Detailed information is listed in Table 1; (**e**) photodiode and phototransistor I-V characteristic with different irradiances at a wavelength of 450 nm; (**f**) the ratio of I-V curves of the photodiode to phototransistor at intensities of 5 mW/cm^2^ and 10 mW/cm^2^. Inset shows an equivalent circuit of phototransistor composed of general photodiode and bipolar junction transistor.

Before starting an in-depth study of the device, it is necessary to compare the NIP PD and NIPIN PTR. First, we used the same geometry of the single PTR cell reported in [2] for our study. For a thorough comparison, we set the same active region and size for the PD and PTR. From the fundamental PTR structure, the NIP PD structure was determined by removing small intrinsic and n+ doping regions, as shown in Figure 1a. Also, for the PTR, we screened a small intrinsic, and n+ doping region using a gold layer for preventing unwanted light absorption in this region [30,31]. The specific geometrical parameters for the NIP photodiode and the NIPIN phototransistor are listed in Table 1. Both devices have a doping depth of 500 nm, and the n+ and p+ regions have a doping concentration of 10^15^ cm^−3^, respectively. In the PTR and PD structures, a finger-type doping region was exploited to reduce the loss of the photo-generated carriers using the side depletion region between the n+ region and the p+ region [32]. This also broadens the active region producingphoto-generated carriers, which increases the cell efficiency. For the operation of PD, the n+ and p+ regions are contacted by an Au electrode, whereas the emitter n+ and collector n+ regions are contact areas of the Au electrode and Si in the PTR. 

For a comparison of the IV characteristics between both devices, these characteristics were simulated at a light intensity of 5 mW/cm^2^ and 10 mW/cm^2^, at a wavelength of 450 nm (Figure 1e). At the same bias point, the phototransistor has a higher photocurrent. Our PTR device is a photo-bipolar junction transistor (BJT) that has a floating base. This floating base and base-collector junction are used as an absorption region for the light. For a photo-BJT in active mode, the holes generated from the absorption region accumulate in the base region. This surplus accumulated charge allows the emitter to inject electrons into the base. Since this mechanism causes an internal gain in the device, the PTR has a higher photocurrent than the PD [33]. Figure 1f shows the difference in I-V performance between the PD and the PTR due to the gain. Because of the gain, the PTR provides a possible solution to the low responsivity of a curved photodetector array owing to the thin film-type structure.

### 2.2. Thickness Simulation

**Figure 2:**
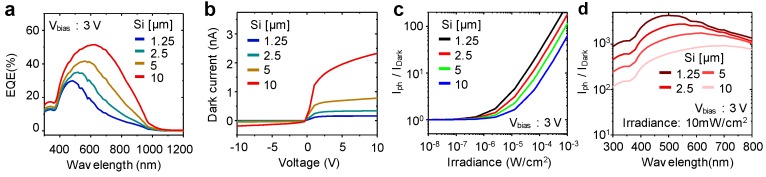
(**a**) External quantum efficiency versus wavelength on various thicknesses at bias voltage of 3 V; (**b**) dark current characteristics of the phototransistor (PTR) as a function of voltage with different thicknesses; (**c**) plots of the ratio of photocurrent to dark current versus the irradiance of light at 3 V and four different Si thicknesses; (**d**) spectral characteristic of the ratio of photocurrent to dark current with variation of Si thickness.

The thickness of the active cell is a major factor in the performance of a photodetector. The characteristics of a photodetector depend strongly on the wavelength because the depth of penetration in Si varies according to the wavelength [34]. In a lateral phototransistor, carriers can be generated more efficiently at a shorter wavelength because the depletion region in the phototransistor is formed near the surface. Also, the thickness of the cell affects the flexibility of the device. For instance, a 1 μm thick Si has a bending curvature of around 10^4^ m*^−^*^1^, whereas a 10 μm thick Si has a bending curvature of around 10^3^ m*^−^*^1^ [25,26]. In this simulation, the doping concentration at the n+ and p+ regions was 10^15^ cm*^−^*^3^ and the thicknesses are 1.25 μm, 2.5 μm, 5 μm, and 10 μm, respectively. Figure 2a shows each EQE with different thicknesses of the phototransistor. As expected, the thicker device has a high EQE due to a long penetration depth in Si for such a long wavelength. However, increasing the thickness not only increases the EQE but also the dark current. The dark current is a noise factor in a photodetector because it is generated regardless of the input light. One of the strong sources of the dark current is thermally generated minority carriers within the bulk-Si region [35]. In addition to thermally generated carriers, for a lateral phototransistor fabricated on a bulk Si substrate, the difference in free carrier concentration between the intrinsic bulk-Si region and the depletion region creates a sharp concentration gradient. This discontinuity of the interface between the depletion and intrinsic Si bulk region causes minority carrier diffusion into the depletion region, which contributes to the dark current. For these reasons, the thicker Si PTR has a higher dark current than a thin PTR, as illustrated in Figure 2b.

An important figure of merit for an image sensor is the linear dynamic range (LDR), which determines the sensitivity of an image sensor. The LDR in the image sensor follows the equation below [36].

Linear dynamic range = 20Log(I_Photo_/I_dark_) dB (1)

where I_dark_ is the dark current and I_Photo_ is the measured photocurrent. Therefore, the ratio of dark current to photo-current should be considered for the use of the image sensor. Figure 2c shows the ratio of dark current to average photo-current with wavelengths of 450, 550, and 650 nm as a function of irradiance. It is seen that the 1.25 μm thick devices have a larger dynamic range than the devices with other thickness values due to the small dark current. Moreover, Figure 2d shows that the Si PTR with a thickness of 1.25 μm has a large dynamic range from the short wavelength range to a long wavelength range. From these results, although the cell should be thin for flexibility, a thin Si PTR can be applied as an image sensor owing to the large dynamic range.

### 2.3. Doping Concentration Simulation

The doping concentration affects the device performance of a phototransistor. The doping concentration determines the width of the depletion region of the intrinsic region, which affects not only light absorption but also the recombination of minority carriers [34]. This also determines the current gain of the internal BJT [37]. Around the depletion region, the main carriers may diffuse into the relative region and can recombine with the main carriers in the other region. In this process, the generated holes and electrons disappear. In the case of high impurity concentration, the number of main carriers increases, enabling recombination with other carriers in a short distance. In other words, the depletion layer becomes thinner. Otherwise, in a semiconductor junction with a low impurity concentration, the depletion layer is thick because the recombination requires a further distance. 

**Figure 3:**
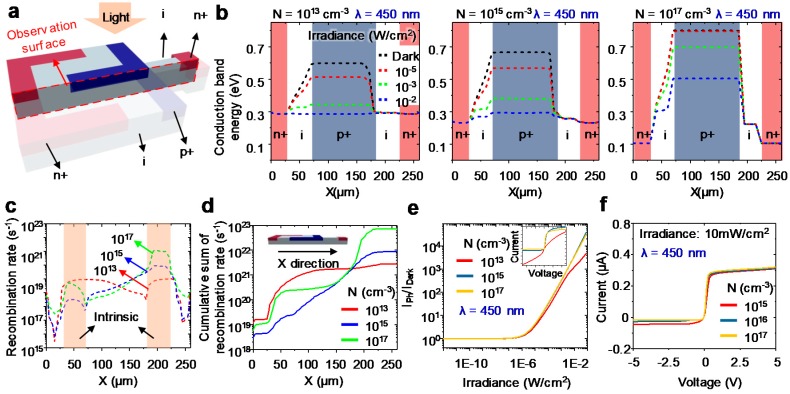
(**a**) 3D scheme of lateral phototransistor and cross-sectional view; (**b**) conduction band profile with different doping concentrations and light intensity along the X direction at the cross-sectional line in Figure 3a; (**c**) recombination rate with three doping concentrations at the cross-sectional plane; (**d**) cumulative sum of recombination rate for different doping concentrations along the X direction; (**e**) photocurrent and dark current ratio for three impurity concentrations as a function of irradiance at a wavelength of 450 nm. The inset presents the I-V characteristics for each concentration at a wavelength of 450 nm and irradiance of 10 mW/cm^2^; (**f**) plot of I-V characteristics for inrinsic silicon having carrier concentration of 10^14^ cm^−3^ with different doping concentrations at a wavelength of 450 nm and irradiance of 10 mW/cm^2^.

To investigate the effects of doping concentration, several simulations were performed on a device that equally doped p+ and n+ regions with a thickness of 1.25 μm. Figure 3b shows the conduction band at zero bias for a cross section of Figure 3a. The incident light generates electron-hole pairs at the base region, which lowers the conduction band of the base region. This result shows the band profiles of the device become different depending on doping concentrations and light intensities at a wavelength of 450 nm. In the case of low doping concentration (i.e., 10^13^ cm^−3^), the conduction band becomes lower as the incident light becomes stronger. Eventually, the conduction band is flattened at irradiance of 10^−2^ W/cm^2^. Contrastively, the device with high doping concentration maintains a conduction band relatively well as the incident light becomes stronger. In the conduction band, the devices with higher doping concentrations keeps the band shape well under strong light illuminations. 

Based on the theoretical analyses with the conduction band, the tendency of PTR was investigated with respect of light intensity and doping concentration. Figure 3c shows the recombination rate for the cross sections of PTR with different doping concentrations. Heavy doping concentration causes a high recombination rate in the intrinsic region owing to the short effective carrier lifetime [38]. Figure 3d shows the integration of the recombination rate along the X direction for each doping concentration. The device with a doping concentration of 10^17^ cm^−3^ demonstrates a higher recombination rate than the other devices; however, it maintains the band shape under strong illumination. The lightly-doped devices no longer show linearity with light intensity over 10^−3^ W/cm^2^ because the characteristics of the BJT are lost (Figure 3e). The dynamic range of the device with the heavy doping concentration maintains linearity. The inset in Figure 3e exhibits the I-V characteristics for each doping concentration. Although the I-V characteristic is slightly degraded compared to that with the doping concentration of 10^15^ cm^−3^, this subtle degradation in the I-V curve is not imperative to operate as a single cell of PTR array. To investigate a practical case, the I-V curves reflecting the carrier concentration of 10^14^ cm^−3^ in the intrinsic region are simulated for the fabrication of PTR (Figure 3f). Considering both linearity and gain for an image sensor, the doping concentration of PTR should be heavier than 10^15^ cm^−3^.

## 3. Fabrication of Phototransistor and Passive Matrix

**Figure 4:**
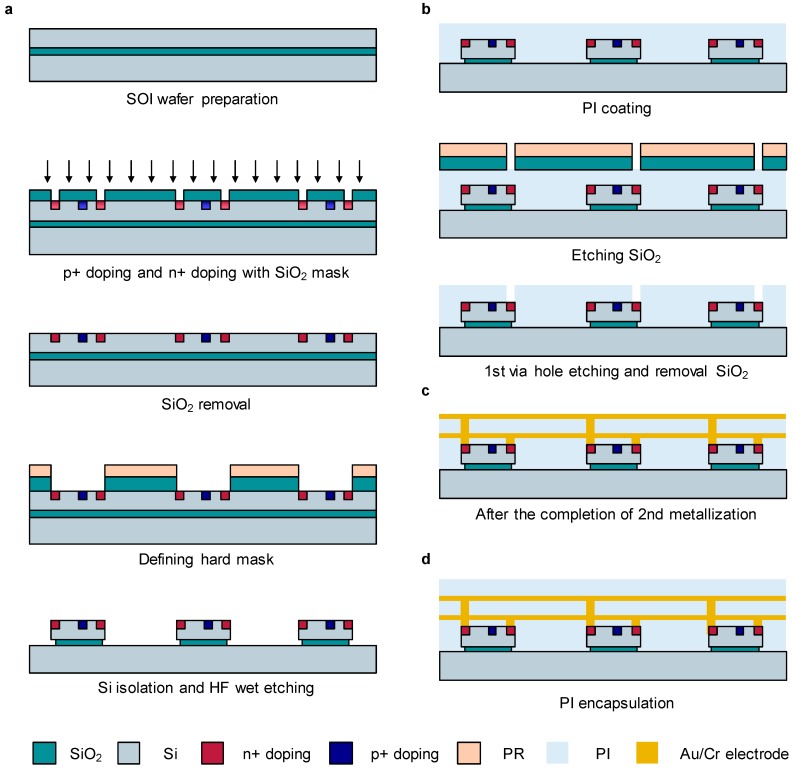
Schematic illustrations of the fabrication process of the curved phototransistor array. (**a**) phototransistor devices; (**b**) etching for metallization process between each pixel; (**c**) Au/Cr electrode metallization; (**d**) polyimide (PI) encapsulation.

The fabrication process of a curved phototransistor array is largely classified into two parts: the manufacture of the PTR and the development of a passive matrix. Figure 4 shows a schematic illustration of the fabrication process for the phototransistor and the wiring of the PTR. We used a silicon on insulator (SOI) wafer with a Si thickness of 1.25 μm, which presented the highest dynamic range, as seen in Figure 2. The thickness of the buried oxide layer is 400 nm. The following steps describe the detailed fabrication process of the phototransistor array.

Step 1: phototransistor pixel defining, as shown in Figure 4a. A SOI wafer is prepared with a cleaning process with acetone, isopropyl alcohol (IPA), and deionized (DI) water. For a doping concentration of 10^15^ cm^−3^, first, a SOI (100) wafer with a patterned SiO_2_ layer is exposed by diffusive boron (PDS BN 1050) at a temperature of 1000 °C for 10 min. Subsequently, phosphorous (PDS PH 1000 N) is diffused on the wafer at a temperature of 1000 °C for 10 min. After n+ and p+ doping, the annealing process is performed to restore crystallinity of Si layer with a temperature of 1000 °C for 20 min. As a hard mask for dry etching, silicon dioxide (SiO_2_) with a thickness of 600 nm is deposited by plasma-enhanced chemical vapor deposition (PECVD). A photoresist (PR; AZ5214, MicroChemicals, Ulm, Germany) is then spin-coated onto the sample with a speed of 3000 rpm for 30 s. Next, photolithographic patterning of the PR is followed by thermally curing the PR (60 s at 90 °C) to define the area of the phototransistor. Additional thermal curing on the sample is conducted to improve the adhesion between the PR and SiO_2_ (2 min at 110 °C). Afterward, the hard mask layer, SiO_2_, is patterned by wet etching with a buffered oxide etchant (BOE) for ~15 s. Next, naked Si is etched by an inductively coupled plasma-reactive ion etcher (ICP-RIE; 4 mTorr, 50 sccm SF_6_, RF 50 W, ICP 100 W, 6 min) to define the area of the device. Finally, the isolated sample is immersed in hydrogen fluoride (HF) solution for ~2 min 30 s to partly etch the edge of SiO_2_ under the Si layer.

Step 2: Polyimide (PI; 431176, Sigma-Aldrich, St. Louis, MO, USA) opening for wiring, as shown in Figure 4b. First, liquid-state PI is spin-coated onto the sample at 4000 rpm for 40 s to form a ~1.2 μm-thick PI layer and then cured at 230 °C for 2 h in an oven under a N_2_ atmosphere. A SiO_2_ layer with a thickness of 300 nm is deposited by PECVD at a relatively low temperature, 230 °C, to avoid thermal damage on PI. Reacting ion etching (RIE; 30 mTorr, 30 sccm O_2_, RF 30 W, 25 min) through a photolithographically patterned hard mask SiO_2_ removed the PI in the open regions. The residual PR and SiO_2_ layers are then removed by acetone and BOE, respectively. 

Step 3: Au/Cr electrode wiring for the passive matrix of phototransistors, as shown in Figure 4c. Before the first metallization, a cleaning process is performed. Cr (~10 nm) is deposited by sputtering followed by Au deposition (~200 nm). The liquid-state PI is spin-coated and thermally cured in the oven. Next, for the second metallization, the same process outlined in Step 2 is performed to open the area for the contact of the electrode. 

Step 4: PI encapsulation, as shown in Figure 4d. For protection of the devices, a third PI layer is formed by spin-coating and a thermal curing process. 

## 4. Characterization of PTR Arrays with Passive Matrix Addressing

**Figure 5:**
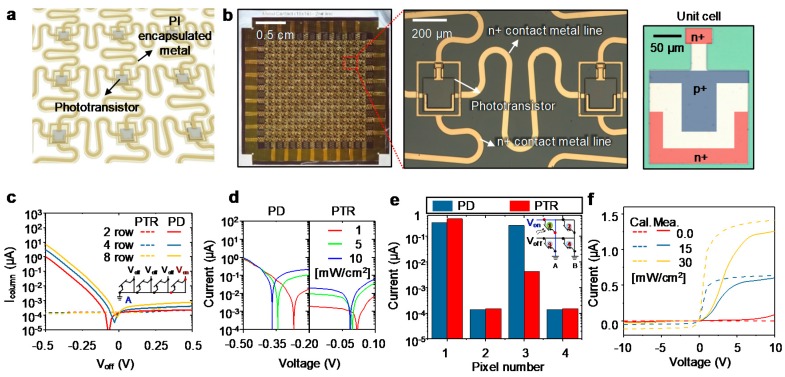
(**a**) Schematic illustration of phototransistor array; (**b**) photograph and microscope image of the fabricated device and the picture on the right side is a magnified unit cell. The colored area indicates the doped regions; (**c**) scheme of phototransistor passive matrix with different numbers of rows. Inset shows the scheme of PTR array with 4 rows; (**d**) plot of current for single photodiode (PD) and PTR with various irradiances as a function of V_off_ at a wavelength of 450 nm; (**e**) Values of current in different phototransistor positions at V_off_ of 0V applied to the non-addressed rows in column A, as displayed in Figure 5c (V_on_ = 3 V). Inset shows 2 by 2 passive matrix which has addressed and non-addressed pixels; (**f**) Plot of I-V curve for simulation and experimental measurement of PTR array.

To evaluate the capability of the proposed PTR array, the characteristics of the I-V curve and crosstalk in the array form should be discussed. To this end, we fabricated a PTR array as a passive matrix type. Figure 5a illustrates the scheme of the complete PTR array. Figure 5b shows the completed PTR array consisting of 16 by 16 phototransistors (i.e., the number of pixels is 256), and magnified views after the second metallization. The unit cell in Figure 5b is the status finished up to the HF wet etching step. When a readout circuit senses the photocurrent of a given pixel, a positive voltage is applied to a row electrode on a column electrode. This bias voltage works as a reverse bias to the pixel located at the intersection of the two electrodes and causes current dominated by the photocurrent or dark current of the diode. For this reason, other pixels in the same column contribute to the dark current or photocurrent of the column current. This crosstalk in the passive matrix is defined as follows [39].

*I_column_ = I_L,D(on)_* + *(N −* 1*)I_L,D(off)_* (2)

where *I_column_* is the current generated from the column displayed as the inset of Figure 5c, *I_L_* is the photocurrent of the illuminated phototransistor, *I_D_* is the dark current of the non-illuminated phototransistor, and *N* is the number of rows. To select the pixel in the passive matrix, the bias voltage is applied to the addressed pixel against the ground, whereas zero voltage is applied to the non-addressed pixel instead of the bias voltage. However, setting zero voltage to the non-addressed pixel is difficult in a real passive matrix device. In real devices, a small voltage (close to zero) is applied to the non-addressed pixels. To investigate the current of the column, we set the voltages applied to the addressed phototransistor and non-addressed phototransistor as V_on_ and V_off_, respectively. As the current from the non-addressed phototransistor is dependent on V_off_, the current influenced by V_off_ is an important factor in array characteristics. 

Figure 5c demonstrates the feature of *I_column_* with different numbers of rows (i.e., different *N* in Equation (2)) for NIP PD and NIPIN PTR in array forms under the dark state. In the case of the NIP PD, the variation of V_off_ varied significantly as *N* increases. Since the illumination light is absent, *I_column_* is composed of dark current from other pixels, indicating the passive matrix of the PD can suffer from serious noise. Moreover, V_min_, indicating the voltage corrsponding to the minimum point of current, is affected by *N*, whereas the *I_column_* of PTR array is unchanged upon voltage variation from −0.5 to 0.5 V. In addition to the unchanged V_min_ point, the NIPIN PTR array is robust to the noise signal from adjacent pixels compared to the NIP PD array. 

To reduce crosstalk in a passive matrix, a single pixel should not have variation in the V_min_ caused by the external light, because the current of the active pixel sensed by the readout circuit could not be stable. Figure 5d shows the effect of the light intensity on the V_min_ of a single PD and the PTR. In the case of the PTR, when the intensity of light changes from 1 mW/cm^2^ to 10 mW/cm^2^, V_min_ varies by about −0.02 V. On the other hand, in the case of the PD, the difference of V_min_ is −0.26 V. For insight into the meaning of V_min_, we compared the current variation at 0 V, 0.2 μA in the PD, and 5 nA in PTR. Therefore, the passive matrix, which consists of PDs, presents substantial noise from the non-addressed pixels in the presence of light owing to the fluctuation of V_min_.

Figure 5e shows the current values of PTR and PD in a two by two passive matrix form. To detect the current in the passive matrix, V_on_ and V_off_ are applied to the addressed rows and non-addressed rows as 3 V and 0 V, respectively. The current values of each pixel are then read. The first pixels of PTR and PD in column A are illuminated with 10 mW/cm^2^, as illustrated in the inset of Figure 5e. Other pixels in columns A and B are not illuminated. However, the current value of the third pixel, which is in column A, is higher than the dark current of the second or fourth pixel, which is in column B, because it was the crosstalk of adjacent pixels in the same column. Comparing the third pixels of PD and PTR, the results demonstrate that the PD has a higher dark current than the PTR. From the results in Figure 5c–e, the passive matrix composing the PTR presents many strengths with respect to crosstalk compared to the passive matrix of the PD.

Figure 5f demonstrates the I-V curve of the fabricated PTR array with different illumination intensities: 0, 15, 30 mW/cm^2^. A xenon lamp with a color temperature of 5800 K (commercial light source SLS401,Thor lab) was utilized as a light source in the measurements. The slope of the curve and the saturated current are different from the calculation results because of differences in the doping profiles (i.e., uniform and Gaussian distributions) and the ideality factor changed by the light intensity [34]. The deviation from the ideality factor and doping profile is acceptable as compared to the ideal case. Also, the tendency of I-V characteristics is akin to each other. Because of this, the simulation results guide the design of PTR for fabrication.

In addition to the electrical performance of the device, the deformability of flexible and/or curved image sensors in array type should be considered. Many other methods for developing the curvilinear shape image sensors based on inorganic materials have been reported (Table 2). Traditionally, a method of bending using a pressure after thinning the thickness of a commercial image sensor has been studied [8,11]. This approach has a strength that the high-resolution imager can be utilized; however, the deformability is seriously restricted by strain. Using inorganic nanomembranes which have flexibility caused by a film-type device is one of the other methods to improve the deformability [40,41,42,43]. However, the 3-dimensional deformation of devices requires stretchability as well. On this basis, the general nanomaterial-based curved/flexible image sensor is difficult to deform into a hemispheric shape. Kirigami- and array-based image sensors can provide high deformability such as a hemispherical shape [1,2,3,17]. However, the kirigami-based image sensor is exactly fabricated to fit with a dome, limiting the curvature variation. Our approach, which is based on array type, shows the highest deformability and radius of curvature. Also, stretchable metal interconnectors between each pixel offer curvature-tunable photodetector [3]. 

## 5. Geometry Optimization

**Figure 6:**
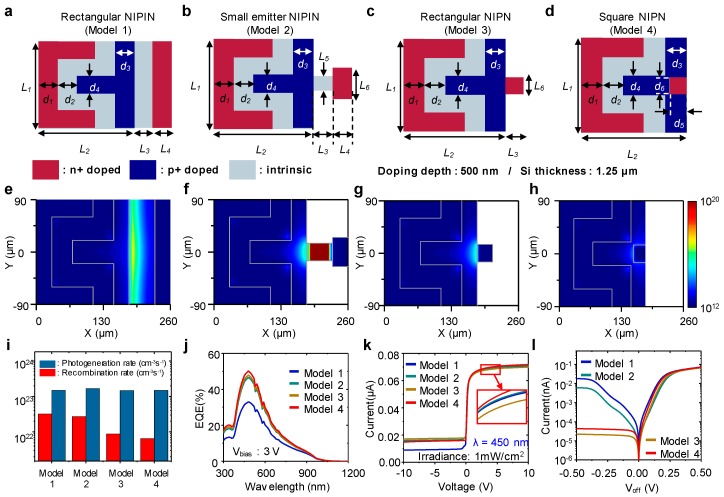
Four different phototransistors with a Si thickness of 1.25 μm and a doping concentration of 10^15^ cm^−3^: (**a**) rectangular NIPIN model; (**b**) small emitter NIPIN model; (**c**) rectangular NIPN model; (**d**) square NIPN model; (**e**–**h**) contour plots, which are positioned at the plane 50 nm from the surface, of recombination rate for each design; (**i**) total recombination rate and photogeneration rate for each model; (**j**) EQE for each model; (**k**) I-V characteristic at the wavelength of 450 nm and irradiance of 1 mW/cm^2^; (**l**) dark current characteristic as a function of V_off_ for each design.

Thus far, we have discussed the advantages and excellence of the PTR as a single cell and even an array type. However, small intrinsic and n+ doping regions in this structure are exposed to the risk of fracture due to a mechanically unstable geometry. Moreover, the small intrinsic region in the PTR is not necessary in principle. Therefore, we designed and simulated four different geometries, including the fundamental structure we have discussed, to find the optimum geometry of the PTR in electrical/mechanical aspects. The detailed geometrical parameters are listed in Table 3. The first design, model 1, is a rectangular NIPIN model, where the intrinsic and n+ doping regions are expanded from the design we have studied (Figure 6a). Model 2 is our basic design, which is effective to reduce the recombination rate from the intrinsic region (Figure 6b). To suppress the recombination rate further, the intrinsic region is removed followed by direct contact with the n+ doping region to the p+ doping region (Figure 6c). In model 4, a n+ doping region is inserted into the p+ doping region, which is the most stable geometry mechanically (Figure 6d).

Figure 6e–h displays the recombination rate in the plane 50 nm from the surface for each model. In models 1 and 2, strong recombination occurs in the intrinsic regions, whereas models 3 and 4, which do not have the intrinsic region, show comparatively weak recombination. Figure 6i presents the total recombination by summation of all recombination rates in the contour plots, and it also gives the total photogeneration rate in the same planes. These results demonstrate the low recombination rates in the designs without intrinsic regions, while similar photogeneration rates are obtained. Model 4 has the highest EQE among all models, as illustrated in Figure 6j. This can be explained by the low recombination rate. Simulations of I-V curves for each model are also conducted, as shown in Figure 6k. In the active range, all devices represent comparable current versus voltage features, but model 1 has a vast amount of current in the range of negative voltage due to the wide intrinsic region. In the array form, the feature of V_off_ is imperative because it is equivalent to the noise signal to the active cell. From this point of view, the devices without the intrinsic region are appropriate as the pixels of a photodetector array, as demonstrated in Figure 6l. With consideration of mechanical and electrical aspects, model 4 is the most promising cell geometry for the curved photodetector array. 

## 6. Conclusions

In this paper, we investigated a lateral NIPIN phototransistor (PTR) array, which was reported in previous research [2], under different conditions such as thickness and doping concentration. We first compared the NIPIN PTR and PIN photodiode (PD) as a pixel of the curved photodetector array. This comparison showed that the NIPIN PTR can be a solution to address low absorption efficiency of the active cell owing to the film-type device. Also, because the thin active cell has an advantage with respect to the dark current, a large dynamic range can be realized compared to that of a thick Si PTR. Also, we have designed and simulated the optimum geometry of the PTR for a curved photodetector array in both mechanical and electrical aspects. The optimized structure (Model 4) in this paper will be experimentally fabricated and demonstrated as future work. We also fabricated the PTR array to examine the validity of our simulations. In the fabrication process, a silicon on insulator (SOI) wafer with an Si thickness of 1.25 μm was doped by a molecular diffusion process of phosphorous and boron at 1000 °C for 10 min. Subsequently, the annealing treatment was performed at 1000 °C for 20 min. The PTR was fabricated through a series of semiconductor fabrication processes including deposition, photolithographical patterning, and etching. 

Thus far, in the field of flexible/wearable electronics, although there has been intensive research on the mechanical/material components of devices, theoretical studies on optimization regarding electronics have not yet been conducted. In this light, the proposed PTR can provide a design rule for high-efficiency Si-based flexible/wearable optoelectronic devices.

## Figures and Tables

**Table 1:** Geometrical parameters for each design (Unit: μm).

Model	*L*_1_	*L*_2_	*L*_3_	*L*_4_	*L*_5_	*L*_6_	*D*_1_	*D*_2_	*D*_3_	*D*_4_
Photodiode	180	180	50	30	-	-	30	40	30	40
Phototransistor	180	180	50	30	30	50	30	40	30	40

**Table 2:** Information of flexible/curved image sensors based on inorganic materials.

Materials	Radius of Curvature	Deformability	Mechanism	Ref.
Si	73 mm	Small	Thinning and bending	[8]
Si	~19 mm	Small	Thinning and bending	[11]
Si	>15 mm	Cylindrical shape	Nanomembrane	[40]
Ge	77.5 to 21 mm	Cylindrical shape	Nanomembrane	[41]
InGaAs	>2.5 mm	Cylindrical shape	Nanomembrane	[42]
Si	28.5 mm	Cylindrical shape	Nanomembrane	[43]
Si	~10 mm	Almost hemisphere	Photodetector array	[1]
Si	>11.4 mm	Almost hemisphere	Photodetector array	[3]
Si	~6.96 mm	Hemisphere	Photodetector array	[2] ^1^
Si	-	Hemisphere	Kirigami	[17]

^1^ The photodetector array is the same structure with this research.

**Table 3:** Geometrical parameters for each design (Unit: μm).

Model	*L*_1_	*L*_2_	*L*_3_	*L*_4_	*L*_5_	*L*_6_	*D*_1_	*D*_2_	*D*_3_	*D*_4_	*D*_5_	*D*_6_
1	180	180	50	30	-	-	30	40	30	40	-	-
2	180	180	50	30	30	50	30	40	30	40	-	-
3	180	180	30	-	-	30	30	40	30	40	-	-
4	180	180	-	-	-	-	30	40	30	40	25	30
